# Supplementary material for: WordCommentsAnalyzer: A windows software tool for qualitative research
Source: F1000Res. 2018 Sep 4;7:536. Originally published 2018 May 3. [Version 2] doi: 10.12688/f1000research.14819.2 (PMC6137411; doi:10.12688/f1000research.14819.2)
Supplement: Supplementary file 3 [file f1000research-7-17614-s0002.tgz › b2a79496-4ada-47c4-9682-a99ce867836d.zip!/Eight Iranian Health Journals/Journal of Research in Rehabilitation Sciences _31.docx]

© Journal of Research in Rehabilitation Sciences . This work is licensed under CC BY-NC 4.0 (https://creativecommons.org/licenses/by-nc/4.0/).

Volume & Issue: دوره 9، شماره 2: 1392

Title: تعیین روایی و اعتبار آزمون اطلاعات آوایی در کودکان 3 تا 6 ساله شهر اصفهان

http://jrrs.mui.ac.ir/index.php/jrrs/article/view/811

مقدمه: اختلال در تلفظ صداها، شایع‌ترین اختلال گفتاری است و شیوع آن در کودکان دبستانی حدود 7/8 درصد می‌باشد. در بحث اختلال در تلفظ صداها، تشخیص و ارزیابی دو عامل مهم می‌باشند. در ارزیابی می‌توان از آزمون‌های رسمی و غیر رسمی استفاده نمود. آسیب‌شناسان گفتار و زبان در ایران برای ارزیابی اختلال در تلفظ صداها به طور وسیعی از آزمون اطلاعات آوایی استفاده می‌کنند که متأسفانه تاکنون اعتبار و روایی این آزمون مشخص نگردیده است. هدف از این مطالعه، ارایه‌ اعتبار و روایی آزمون اطلاعات آوایی بود تا سرآغازی برای استاندارد شدن این آزمون باشد. مواد و روش‌ها: مطالعه حاضر از نوع توصيفي و روش‌شناسی بود. روايي محتوايي آزمون با اعلام نظر 20 آسيب‌شناس گفتار و زبان تعیین گردید. پس از این مرحله آزمون بر روی 150 کودک 3 تا 6 ساله اجرا و براي تعيين اعتبار آزمون از دو روش 20Kuder-Richardson  و بازآزمایی استفاده شد. تجزیه و تحلیل داده‌ها توسط نرم‌افزار SPSS نسخه 13 صورت گرفت. يافته‌‌‌ها: مقدار CVR (Content validity ratio) براي واژه‌ها به استثنای /kæʧæl/ بالاتر از 42 درصد بود. نتایج حاصل بر اساس روش 20Kuder-Richardson  و ضریب همبستگی در هر 3 گروه سنی نشان داد که آزمون اعتبار لازم را دارا می‌باشد. نتيجه‌گيري: نتايج حاصل از اين پژ‍وهش نشان داد كه آزمون اطلاعات آوایی از اعتبار و روايي لازم برخوردار است. در حقیقت آزمون دقت و صحت کافی دارد تا بتوان از آن در تشخیص و ارزیابی استفاده نمود.

Title: تأثیر برنامه تمرینی ایروبیک و بازی درمانی گروهی بر تعادل کودکان مبتلا به اختلال نقص توجه و بیش‌فعالی

http://jrrs.mui.ac.ir/index.php/jrrs/article/view/903

مقدمه: اختلال نقص توجه و بیش‌فعالی نوعی اختلال رشدی است. کودکان مبتلا به اختلال نقص توجه و بیش‌فعالی به طور عمده با هماهنگی حرکتی ضعیف و دست و پاچلفتی توصیف می‌شوند. به نظر می‌رسد تمرینات حرکتی ریتمیک مانند بازی‌های حرکتی نیز می‌تواند بر تعادل کودکان مبتلا به این اختلال تأثیرگذار باشد. هدف این پژوهش، مقایسه تأثیر برنامه تمرینی ایروبیک و بازی درمانی گروهی بر تعادل کودکان مبتلا به اختلال نقص توجه و بیش‌فعالی بود. مواد و روش‌ها: در پژوهش حاضر، 26 کودک (4 دختر و 22 پسر) مبتلا به اختلال نقص توجه و بیش‌فعالی در 3 گروه تجربی (بازی درمانی گروهی و ایروبیک) و شاهد مورد مطالعه قرار گرفتند. گروه‌های تجربی هر یک 8 هفته، هر هفته 3 جلسه 60-45 دقیقه‌ای، تحت مداخله قرار گرفتند. تعادل استاتیک و تعادل داینامیک هر سه گروه به ترتیب با تست تعادل Flamingo (Flamingo balance test) و تست تعادل ستاره‌ای مورد ارزیابی قرار گرفت. یافته‌ها: بین گروه بازی درمانی گروهی و ایروبیک در تعادل پویا تفاوت معنی‌داری وجود داشت، اما در تعادل استاتیک تفاوت معنی‌داری بین گروه‌های تحت مداخله مشاهده نشد. نتیجه‌گیری: با این‌که بازی درمانی گروهی به طور کلی تأثیر بیشتری بر بهبود تعادل کودکان مبتلا به نقص توجه و بیش‌فعالی داشت، اما ایروبیک نیز در بهبود تعادل ایستا تأثیر معنی‌داری داشت. بنابراین استفاده از تمرینات ایروبیک در کنار بازی درمانی گروهی می‌تواند باعث بهبود بیشتر تعادل این کودکان گردد.

Title: تأثیر تمرینات مقاومتی با و بدون تحریکات الکترومغناطیس پالسی بر قدرت عضلانی و تعادل بیماران هموفیلی شدید A

http://jrrs.mui.ac.ir/index.php/jrrs/article/view/865

مقدمه: افزایش قدرت عضله و انجام ورزش‌های مقاومتی همواره به عنوان یکی از راه‌های بالقوه برای بهبود تعادل در نظر گرفته شده است. در این مطالعه اثر تمرینات مقاومتی با و بدون میدان الکترومغناطیس پالسی بر قدرت عضلانی و تعادل بیماران هموفیلی شدید A بررسی شد. مواد و روش‌ها: 32 بیمار هموفیلی شدید A (18 تا 35 ساله) به صورت تصادفی در سه گروه تمرین مقاومتی، تمرین مقاومتی توأم با میدان الکترومغناطیسی پالسی (مگنت) (Pulsed electromagnetic field یا PEMF) و شاهد قرار گرفتند. گروه تمرین مقاومتی (40-30 دقیقه تمرینات مقاومتی تنه و اندام‌ها با 60-50 درصد 1RM (Repetition maximum) (تکرار بیشینه) به صورت پیش‌رونده و 30 دقیقه میدان مگنت به صورت دارونما) و گروه تمرین مقاومتی توأم با مگنت، تمرینات با همان شدت، ولی تکرار کمتر و سپس 30 دقیقه تحریک PEMF با فرکانس 30 هرتز و شدت 40 گوس، 6 هفته و 3 جلسه در هفته درمان شدند. در گروه شاهد هیچ تداخلی انجام نشد. میزان قدرت عضلانی بر حسب 1RM و آزمون‌های تعادل (One leg standing، Near tandem stand، Timed up and go و Timed sit to stand) قبل و بعد از 6 هفته اندازه‌گیری گردید. يافته‏ها: انجام 6 هفته تمرین مقاومتی و تمرین مقاومتی توأم با PEMF، باعث بهبودی معنی‌دار آزمون‌های تعادلی و قدرت عضلانی نسبت به گروه شاهد و مقادیر پایه شد. نتيجه‏گيري: انجام تمرینات مقاومتی با و بدون PEMF در افزایش قدرت عضلانی و تعادل بیماران هموفیلی مؤثر است.

Title: تاثیر نوروفیدبک بر عملکرد تعادلی و توجه انتقالی کودکان مبتلا به اختلال خواندن

http://jrrs.mui.ac.ir/index.php/jrrs/article/view/666

مقدمه: اختلال خواندن جزء اختلالات عصبی- رشدی با نقایصی در مهارت‌های شناختی و حرکتی است. نوروفیدبک به عنوان یک روش درمانی جدید، با اصلاح ناهنجاری‌های موجود در نوار مغزی این کودکان می‌تواند به بهبود عملکرد آن‌ها کمک کند. هدف پژوهش حاضر، بررسی تأثیر پروتکل تعادلی نوروفیدبک بر عملکرد تعادلی و توجه انتقالی این کودکان بود. مواد و روش‌ها: این پژوهش در چارچوب یک طرح تک آزمودنی طی 20 جلسه روی 4 کودک (1 دختر و 3 پسر) 12-8 ساله با اختلال خواندن اجرا شد و تعداد جلسات درمانی در این مطالعه 12 جلسه (30 دقیقه‌ای) بود. برای جمع‌آوری اطلاعات در سطح عملکرد تعادلی از خرده آزمون Bruininks- Oseretsky و در سطح توجه انتقالی از آزمون Posner paradigm استفاده شد. یافته‌ها: اندازه اثر درمان در عملکرد تعادلی برای تمام آزمودنی‌ها به جز اندازه اثر خط پایه- درمان در آزمودنی سه، بالا (8/0 < Cohen’d) بود و در مورد توجه انتقالی نیز درمان تنها در برخی از موارد تأثیر داشت. نتیجه‌گیری: نتایج مطالعه حاضر نشان داد که پروتکل تعادلی نوروفیدبک می‌تواند در بهبود عملکرد تعادلی و تا حدودی در توجه انتقالی کودکان مبتلا به اختلال خواندن مؤثر باشد.

Title: اثر تداخلی دو نوع تکلیف توجهی بر عملکرد تعادلی

http://jrrs.mui.ac.ir/index.php/jrrs/article/view/761

مقدمه: بسیاری از فعالیت‌های زندگی روزانه علاوه بر نیازهای تعادلی نیازهای شناختی نیز دارند. هدف از مطالعه حاضر، مقایسه تأثیر تکلیف توجه انتقالی و انتخابی بر تعادل در جوانان بود. مواد و روش‌ها: در این مطالعه مقطعي، 30 نفر از دانشجویان پسر با دامنه سنی 18 تا 26 سال که با روش نمونه‌گیری در دسترس انتخاب شده بودند، آزمون تعادل یک پا را در 3 حالت تکلیف تعادل تنها، تکلیف تعادل به همراه توجه انتقالی و تکلیف تعادل به همراه توجه انتخابی بر روی دستگاه بایودکس انجام دادند. آزمون استروپ (Stroop) برای سنجش توجه انتخابی و آزمون عصب‌شناختی انتقال توجه برای جمع‌آوری داده‌ها استفاده شد. برای تحلیل از آزمون تحلیل واریانس با اندازه‌های تکراری استفاده گردید. کلیه محاسبات آماری با استفاده از نرم‌افزار SPSS نسخه 16 انجام گرفت. یافته‌ها: یافته‌های حاصل از تحلیل داده‌ها، افت معنی‌دار (05/0 > P) عملکرد تعادلی ایستادن بر روی یک پا را در تکلیف دوگانه در سطوح سهمی و پیشانی و در حالت کلی، به همراه تکلیف ثانویه‌ توجه انتخابی و توجه انتقالی نشان داد. همچنین تکالیف شناختی در شرایط تکلیف منفرد و دوگانه مقایسه شدند. در هنگام مقایسه تکلیف توجه انتخابی و انتقالی در شرایط تکلیف دوگانه نسبت به تکلیف منفرد، افت عملکرد شناختی (05/0 > P) مشاهده شد. مقایسه نتایج نشان داد که با وجود افت عملکرد تکلیف تعادلی در هنگام انجام تکلیف تعادلی هم‌زمان با تکالیف شناختی توجه انتخابی و انتقالی، بین افت عملکرد تعادلی در وضعیت‌های ترکیب شده با توجه انتقالی و توجه انتخابی تفاوت معنی‌داری دیده نشد. نتیجه‌گیری: ساختارهای مغزی یکسان درگیر در توجه انتخابی و انتقالی و ارتباط نزدیک بین جنبه‌های مختلف توجه را می‌توان دلیل عدم تفاوت بین افت عملکرد تعادلی در وضعیت‌های ترکیب شده با توجه انتقالی و توجه انتخابی دانست.

Title: بررسی تأثیر یک برنامه تمرینی بر اصلاح پای چرخش یافته به داخل (مطالعه مقدماتی)

http://jrrs.mui.ac.ir/index.php/jrrs/article/view/764

مقدمه: پای چرخش یافته به داخل یکی از ناهنجاری‌های رایج می‌باشد که با افتادگی استخوان ناوی و افزایش چرخش داخلی ران همراه بوده و ممکن است متأثر از عضلات ران باشد. هدف این تحقیق، بررسی اثر یک برنامه تمرینی بر اصلاح پای چرخش یافته به داخل بود. مواد و روش‌ها: در مطالعه حاضر 30 نفر آزمودنی مرد به صورت هدفمند (سن 07/2 ± 63/21 سال، وزن 62/7 ± 93/71 کیلوگرم و قد 52/7 ± 33/177 سانتی‌متر) که ناهنجاری پای چرخش یافته به داخل داشتند انتخاب و به صورت تصادفی در دو گروه 15 نفری شاهد و تجربی قرار گرفتند. با آزمون افتادگی استخوان ناوی، پای چرخش یافته به داخل آزمودنی‌ها اندازه‌گیری و افرادی که دارای افتادگی ناوی بیش از 10 میلی‌متر بودند وارد مطالعه گردیدند و میزان چرخش داخلی ران با گونیامتر اندازه‌گیری و ثبت گردید. همچنین قدرت عضلات ابداکتور و چرخاننده خارجی ران با دیناموتر دستی اندازه‌گیری شد. سپس گروه تجربی، شش هفته و با تواتر سه جلسه در هفته برنامه تمرین قدرتی بر عضلات مذکور را اجرا نمودند و گروه شاهد فعالیت معمول خود را سپری نمود. بعد از اتمام 6 هفته، افتادگی استخوان ناوی آزمودنی‌ها، میزان چرخش داخلی ران و قدرت عضلات دوباره اندازه‌گیری شد. تحلیل اطلاعات با استفاده از آزمون‌های Independent t و Paired t برای اختلافات درون و بین گروهی اجرا گردید (05/0 £ P). یافته‌ها: در گروه تجربی با افزایش معنی‌دار قدرت عضلات ران، میزان چرخش داخلی ران و افتادگی استخوان ناوی آزمودنی‌ها کاهش یافت، اما در گروه شاهد تغییرات معنی‌داری مشاهده نگردید. در مقایسه، گروه تجربی کاهش معنی‌داری در میزان چرخش داخلی و پای چرخش یافته به داخل نسبت به گروه شاهد داشت. نتیجه‌گیری: نتایج تحقیق بیانگر این بود که با افزایش قدرت عضلات ابداکتور و چرخاننده خارجی ران و با استفاده از مداخله تمرینی می‌توان از شدت پای چرخش یافته به داخل پیشگیری نموده و آن را اصلاح نمود.

Title: اثربخشی فعالیت‌های یکپارچگی حسی- حرکتی بر نشانه‌های اختلال نقص توجه- بیش‌فعالی

http://jrrs.mui.ac.ir/index.php/jrrs/article/view/879

مقدمه: ‌اختلال نقص توجه- بیش‌فعالی اختلالی شایع در دوران کودکی است و یافتن درمان مؤثري که از شدت و عمق نشانه‌هاي آن بکاهد، از اهمیت ویژه‌ای برخوردار است. در پژوهش حاضر، اثر یکپارچگی حسی- حرکتی با تأکید بر حواس عمقی و دهلیزی به عنوان یک روش درمانی برای بهبود نشانه‌های اصلی این اختلال مورد بررسي قرار گرفت. مواد و روش‌ها: پژوهش حاضر از نوع مطالعات شبه آزمایشی با پیش‌آزمون و پس‌آزمون همراه با دو گروه آزمایش و شاهد بود. از بین دانش‌آموزان 6 تا 12 ساله مبتلا به اختلال نقص توجه- بیش‌فعالی که در سال تحصیلی 91-1390 در شهر سمنان مشغول به تحصیل بودند، 32 دانش‌آموز به صورت تصادفی انتخاب و به دو گروه 16 نفری آزمایش و شاهد تقسیم شدند. گروه آزمایش هفته‌ای دو بار و طی 12 جلسه دو ساعته، مداخله یکپارچگی حسی- حرکتی (با تأکید بر حواس عمقی و دهلیزی) را دریافت نمودند. نشانه‌های اختلال نقص توجه- بیش‌فعالی هر دو گروه با استفاده از پرسش‌نامه نشانه‌های مرضی کودکان (Child symptom inventory) (4) ارزیابی شد. داده‌های جمع‌آوری شده با آزمون‌های آماری Shapiro-Wilk و تحلیل کوواریانس چند متغیری (MANOVA) و تک متغیری (UNIANCOVA) بررسی شدند. یافته‌ها: طبق آزمون مبتنی بر گزارش معلمان، گروه آزمایش در نمرات متغیر بی‌توجهی و بیش‌فعالی نسبت به گروه شاهد عملکرد بهتری داشت (001/0 > P)، ولی کاهش نمرات متغیر تکانشگری گروه آزمایش نسبت به گروه شاهد (05/0 < P) معنی‌دار نبود، ولي بر اساس آزمون مبتنی بر گزارش والدین در نمرات متغیر بی‌توجهی، بیش‌فعالی و تکانشگری گروه آزمایش نسبت به گروه شاهد عملکرد بهتری داشت (001/0 > P). نتیجه‌گیری: در مجموع مداخله یکپارچگی حسی- حرکتی با تأکید بر حواس عمقی و دهلیزی به طور معنی‌داری نقص توجه، بیش‌فعالی و تکانشگری را در جامعه مورد بررسي بهبود داد.

Title: ارتباط بین سطح فعالیت برخی از عضلات اندام تحتانی با نیروی برشی قدامی زانو در حرکت پرش- فرود تک پا

http://jrrs.mui.ac.ir/index.php/jrrs/article/view/964

چكيده مقدمه:شناسایی فاکتورهایی که باعث شیوع بالای آسیب ACL می‌شود از اهمیت زیادی برخوردار است. نیروهای برشی قدامی مکانیزم اصلی بار وارده به ACL هستند. فعالیت عضلات و پوزیشن اندام تحتانی طی فعالیت می‌تواند باعث افزایش یا کاهش نیروی برشی قدامی شود. هدف از این مطالعه بررسی ارتباط بین سطح فعالیت برخی از عضلات اندام تحتانی با نیروی برشی قدامی در حرکت پرش- فرود تک‌پا بود. مواد و روش‌ها:20 دانشجوي پسر رشته تربیت بدنیدر اين مطالعه شركت كردند. سطح فعالیت الکترومیوگرافی عضلات سرینی میانی، ساقی قدامی و همسترینگ خارجی با استفاده از دستگاه الکترومیوگرافی، و نیروی عکس‌العمل زمین با استفاده از دستگاه صفحه نيرو در حركت پرش-فرود تک‌پا ارزیابی شد. به منظور تجزیه و تحلیل داده‌ها، از آزمون همبستگی پیرسون استفاده شد. يافته‌ها:‌یافته‌ها نشان داد که بین سطح فعالیت عضله ساقی قدامی و سرینی میانی با نیروی برشی قدامی ارتباط معنی‌داری وجود ندارد (05/0P>)، اما ارتباط معنی‌داری بین سطح فعالیت عضله همسترینگ خارجی با نیروی برشی قدامی یافت شد (05/0P<). نتيجه‌گيري: یافته‌های این مطالعه نشان داد که فعالیت عضله همسترینگ در حین حرکت پرش- فرود تک‌پا به عنوان عضله حمایت‌کننده از ACL عمل می‌کند و افزایش فعالیت این عضله می‌تواند منجر به کاهش بار وارده بر ACL شود.

Title: شاخصهای بیومتریکی مرتبط با آسيب ورزشکاران نخبه رشتههای رزمی و ارائه راهكارهاي پيشگيرانه

http://jrrs.mui.ac.ir/index.php/jrrs/article/view/861

چکیده مقدمه: ورزشکاران ملی سرمایه‌ی اجتماعی هستند و آسیب ورزشی واقعیتی است که این سرمایه‌های ملی را تهدید می‌ماید. هدف از پژوهش حاضر، تعيين شاخص‌های بیومتریکی مرتبط با شيوع آسيب ورزشکاران نخبه رشته‌های رزمی و ارائه راهكارهاي پيشگيرانه بود.  مواد و روش‌ها: تحقیق حاضر ارتباط‌سنجي و از نوع همبستگی است. 36 ملی‌پوش مرد رشته‌های کاراته، جودو و ووشو در تحقیق حضور یافتند. ويژگي‌هاي بیومتریکی طبق دستورالعمل  انجمن بین‌المللی پیکرسنجی (ISAK) ارزیابی شد و برای جمع‌آوری اطلاعات آسیب‌ها از پرسشنامه استاندارد آسیب طب ورزشی استرالیا استفاده گردید. براي تعيين شاخص‌های  بیومتریکی مرتبط با آسيب ورزشکاران از آزمون همبستگی پیرسون با استفاده از نرم‌افزار آماری SPSS نسخۀ16 انجام شد.  يافته‌ها: از میان 17 شاخص بيومتريكي مورد مطالعه بين درصد چربی بدن (05/0p<)، جزء اندومورفی نوع‌پیکری (05/0p<)،  شاخص بازويي- زند اعلايي (05/0p<) و طول اندام تحتانی (05/0>p)  با شیوع آسیب‌ها رابطه معنی‌داری  وجود داشت.  نتيجه‌گيري: شرايط حرفه‌اي این ورزشكاران، كاهش‌وزن­ و وزن‌گيري‌هاي متعدد آن‌ها و نقش بافت‌چربي در كاهش توان عضلاني و بروز خستگي عضلاني؛ مي‌تواند تفسيرگر ارتباط درصد چربی بدن و جزء اندومورفی با آسيب ورزشكاران باشد. ارتباط شاخص بازويي-زنداعلايي و طول اندام تحتانی با شیوع آسیب  ورزشكاران رشته‌هاي رزمي ممکن است به فراوانی مهارت‌های دستکاری و نقش توأمان اندام فوقاني و تحتاني در اجراي موفق ورشکاران این رشته مربوط باشد. بر این اساس توجه به برنامه‌هاي آماده‌سازي اختصاصي ورزشكاران در الگوهاي حركتي، عضلات و مفاصل درگير و روش‌های صحیح گاردگیری، ورود ضربه، دفاع و  فرود آمدن پس از پرتاب شدن از کاربردی‌ترین راهبردهای پیشگیرانه است.

Title: بررسی تأثیر استفاده هم‌زمان از تمرینات تکلیف محور و روش محدودیت درمانی اجباری بر روی عملکرد حرکتی اندام فوقانی بیماران همی‌پلژی بزرگسال

http://jrrs.mui.ac.ir/index.php/jrrs/article/view/902

مقدمه: سکته مغزی یکی از شایع‌ترین بیماری‌های نورولوژیک تهدید کننده زندگی و مهم‌ترین علت ناتوانی افراد مسن است. بروز آسیب حرکتی در یک اندام فوقانی به عنوان اصلی‌ترین عامل وابستگی در فعالیت‌های روزمره زندگی به شمار می‌آید. هدف از این پژوهش، بررسی تأثیر استفاده هم‌زمان از تمرینات تکلیف محور و روش محدودیت درمانی اجباری بر روی عملکرد حرکتی اندام فوقانی بیماران همی‌پلژی بزرگسال بود. مواد و روش‌ها: پژوهش حاضر از نوع مداخله‌ای بود و نمونه‌گیری به صورت در دسترس از کلینیک‌های کاردرمانی و مراکز توان‌بخشی استان قزوین انجام شد. 26 بیمار 65-40 ساله سکته مغزی انتخاب شده و 3 روزدر هفته به مدت 8 هفته یک ساعت در روز تمرینات تکلیف محور هم‌زمان با 5 ساعت محدود کردن اندام سالم را انجام دادند. ابزارهای ارزیابی مورد استفاده شامل آزمون‌های Fugl-Meyer و Action research arm به منظور بررسی عملکرد حرکتی اندام فوقانی بود. آزمون آماری Paired t جهت تحلیل داده‌ها مورد استفاده قرار گرفت. از نرم‌افزار SPSS نسخه 18 برای آنالیز داده‌ها استفاده گردید و سطح معنی‌داری 05/0در نظر گرفته شد. یافته‌ها: ارزیابی‌ها نشان دهنده بهبود معنی‌دار عملکرد اندام فوقانی (05/0 > P) بعد از استفاده هم‌زمان از تمرینات تکلیف محور و روش محدودیت درمانی بود. نتیجه‌گیری: نتایج حاصل از تحقیق حاضر نشان داد که استفاده هم‌زمان از تمرینات تکلیف محور روش محدودیت درمانی می‌تواند روش مؤثری جهت بهبود عملکرد اندام فوقانی بیماران سکته مغزی باشد.

Title: برتری جانبی در انتقال نامتقارن قدرت و سرعت در کودکان: با توجه به نقش نیم‌کره‌های مغزی

http://jrrs.mui.ac.ir/index.php/jrrs/article/view/961

مقدمه: در این مطالعه نیمه تجربی، هدف از تحقیق حاضر، مقایسه برتری جانبی در انتقال دو سویه قدرت و سرعت در کودکان 11 تا 13 ساله بود. مواد و روش‌ها: آزمودنی‌ها شامل 28 کودک (62/0 ± 6/11 سال) بود كه از بین مدارس راهنمایی شهرستان کاشمر، انتخاب شدند و در 2 گروه 14 نفره قرار گرفتند. یک گروه با دست راست تمرین قدرتی و با دست چپ تمرین سرعتی و گروه دیگر با دست چپ تمرین قدرتی و با دست راست تمرین سرعتی انجام دادند. آزمودنی‌ها به مدت 4 هفته تمرین کردند که هر هفته شامل 3 جلسه بود. یافته‌ها: نتایج آزمون تحلیل واریانس یک طرفه نشان داد که در میزان انتقال قدرت بین گروه‌ها تفاوت معنی‌داری وجود داشت. میزان انتقال قدرت از دست چپ به راست بیشتر از میزان انتقال قدرت از دست راست به چپ بود  (023/0 = P). با وجود این، در متغیر سرعت تفاوت معنی‌داری وجود نداشت. نتیجه‌گیری: با توجه به نتایج تحقیق حاضر و تخصص نیمکره راست در کنترل قدرت، به نظر می‌رسد که میزان انتقال قدرت از دست چپ به راست بیشتر از میزان انتقال از دست راست به چپ می‌باشد.

Title: رابطه نتایج آزمون‌های سنتی و اصلاح شده نشستن و خمش به جلو با برخی متغيرهاي آنتروپومتريک در دانش‌آموزان پسر مقطع راهنمایی

http://jrrs.mui.ac.ir/index.php/jrrs/article/view/728

مقدمه: آزمون‌های سنتی و اصلاح شده نشستن و خمش به جلو به طور رایجی برای ارزیابی انعطاف‌پذیری کمر و همسترینگ مورد استفاده قرار می‌گیرند. هدف از انجام پژوهش حاضر، بررسی رابطه بین نتایج آزمون‌های سنتي و اصلاح شده نشستن و خمش به جلو با برخی متغيرهاي آنتروپومتريک در دانش‌آموزان پسر مقطع راهنمایی بود. مواد و روش‌ها: 92 دانش‌آموز (با میانگین سنی 67/5 ± 22/13 سال) به عنوان آزمودني در اين تحقيق شرکت کردند. متغیر‌های آنتروپومتریک وزن، طول قد، طول اندام تحتاني، طول تنه و نسبت طول اندام تحتاني به بالاتنه اندازه‌گيري شد. سپس آزمودني‌ها، آزمون‌های سنتی و اصلاح شده نشستن و خمش به جلو را اجرا کردند. براي تجزيه و تحليل داده‌ها از رگرسيون خطي برای محاسبه ضريب تعيين و ضريب استاندارد B استفاده شد. یافته‌ها: ضريب تعيين به دست آمده براي آزمون سنتي (081/0) کمتر از آزمون اصلاح شده (179/0) بود. همچنين طول بالاتنه، بيشترين تأثير و قد، کمترين تأثير را بر نتايج آزمون سنتي (24/1 = B و 80/0 = B) و اصلاح شده (61/2 = B و 82/1- = B) انعطاف‌پذيري داشتند. نتیجه‌گیری: اين يافته‌ها نشان داد که نتايج هر دو آزمون سنتي و اصلاح شده نشستن و خمش به جلو، با برخی از متغيرهاي آنتروپومتريک رابطه دارند. بنابراین، در هنگام استفاده از آن‌ها باید تفاوت‌های فردی را مورد توجه قرار داد.

Title: بررسی اثر ناتوانی بر میزان مواجهه با موانع محیطی مشارکت در کودکان فلج مغزی

http://jrrs.mui.ac.ir/index.php/jrrs/article/view/790

مقدمه: فلج مغزی شامل ناهنجاری‌های غیر پیش‌رونده به مغز در حال رشد است كه منجر به بروز دسته‌ای از مشكلات حركتی می‌شود. علاوه بر مشكلات حركتي كه بر ميزان مشاركت كودك فلج مغزي تأثيرگذار است، عوامل محيطی نيز مي‌تواند ميزان مشاركت را كاهش يا افزايش دهد. هدف این مطالعه، بررسی اثر ناتوانی بر میزان مواجهه با موانع محیطی بود. مواد و روش‌ها: 75 نفر از والدین کودکان فلج مغزی 12-5 ساله در دو مرکز توان‌بخشی تهران به صورت در دسترس به تکمیل پرسش‌نامه عوامل محیطی بیمارستان Craig (Craig hospital inventory of environmental factors یا CHIEF) و پرسش‌نامه ویژگی‌های فردی پرداختند. محقق با گرفتن اطلاعات از والدین به تعیین سطح توانایی دستی، عملکرد حرکتی و سطح شناختی کودک پرداخت. یافته‌ها: والدین کودکان فلج مغزی بیشترین میزان مواجهه این کودکان را با موانع به ترتیب در خرده مقیاس سرویس‌ها، کمک و سیاست‌ها گزارش نمودند. مقایسه نمره خرده مقیاس‌ها و نمره کل از نظر سطح شناختی، توانایی دستی و عملکرد حرکتی درشت کودک تفاوت معنی‌داری را نشان داد. نتیجه‌گیری: موانع محیطی متعددی برای مشارکت کودکان فلج مغزی وجود دارد و با کاهش عملکرد کودک میزان مواجهه با این موانع بیشتر خواهد بود. اصلاح موانع محیطی موجود و فراهم نمودن تسهیلات متناسب با معلولیت کودکان برای مشارکت آن‌ها ضروری است.

Title: اثر کوتاه مدت کفی طبی بر فعالیت الکتریکی و هم انقباضی عضلات ساق افراد دچار صافی کف پا

http://jrrs.mui.ac.ir/index.php/jrrs/article/view/762

مقدمه: عوامل ساختاری قادر هستند روی عملکرد پای انسان اثرگذار باشند. هدف از این مطالعه، تعیین اثر آنی کفی طبی بر میزان فعالیت الکتریکی و هم‌انقباضی عضلات ساق افراد دچار صافی کف پا در مقایسه با گروه شاهد بود. مواد و روش‌ها: تعداد 30 نفر دانشجوی پسر در دو گروه دچار صافی کف پا و شاهد جای گرفته و در این مطالعه شرکت کردند. از الکترومایوگرافی سطحی برای اندازه‌گیری فعالیت عضلات درشت‌نئی قدامی، نازک‌نئی طویل، دو قلوی داخلی و نعلی استفاده شد. آزمودنی‌ها مسیر 15 متری آزمایش را 3 بار در حالت پابرهنه راه رفتند. آزمودنی‌های گروه دچار صافی کف پا علاوه بر حالت پا برهنه در وضعیت‌های تنها با کفش و کفش همراه با کفی طبی مسیر را طی کردند. یافته‌ها: یافته‌های این پژوهش نشان داد که افراد دچار صافی کف پا میزان هم‌انقباضی جهت‌دار بیشتری در مراحل میداستانس (001/0 = P) و پروپالژن (003/0 = P) از خود نشان می‌دهند. استفاده از کفی باعث افزایش میزان هم‌انقباضی در مرحله تماس پاشنه (009/0 = P) و کاهش میزان هم‌انقباضی در مرحله میداستانس (015/0 = P) شد. نتیجه‌گیری: کفی طبی می‌تواند از فعالیت اضافی عضلات جلوگیری کند. همچنین با توجه به اثر کفی بر میزان هم‌انقباضی در مرحله تماس پاشنه، می‌توان حرکت اضافی پاشنه در این مرحله به منشأ غیر عضلانی نسبت داد.

Title: بررسي تأثير هشت هفته تمرینات پیلاتس بر عملکرد حرکتی و افسردگي در بيماران مبتلا به مالتیپل اسکلروزیس

http://jrrs.mui.ac.ir/index.php/jrrs/article/view/669

مقدمه: افسردگی می‌تواند واکنشی درون‌زاد یا قسمتی از بیماری مالتیپل اسکلروزیس (Multiple sclerosis یا MS) باشد. همچنین در اغلب موارد، این بیماری باعث درگیر شدن عضلات می‌شود. بنابراین اكثر این افراد قدرت عضلاني اندام‌های خود را از دست مي‌دهند. بنابراین تحقيق حاضر با هدف، اثربخشی 8 هفته تمرينات پيلاتس بر عملکرد حرکتی و افسردگی در بیماران مبتلا به MS انجام شد. مواد و روش‌ها: تحقيق حاضر از نوع نيمه تجربي با طرح پيش‌آزمون- پس‌آزمون و گروه شاهد بود. شرکت کنندگان در اين تحقيق 33 زن مبتلا به MS [با میانگین سنی: 447/9 ± 36/33 سال و میانگین EDSS (Expanded disability status scale): 278/1 ± 89/2] بودند که در سال 1390 به انجمن MS استان اصفهان مراجعه کردند. شرکت کنندگان به طور تصادفی به دو گروه تجربي (18 نفر) و شاهد (15 نفر) تقسيم شدند. از پرسش‌نامه افسردگي Beck (Beck depression inventory) به منظور ارزيابی افسردگی و از آزمون‌های 30 ثانیه نشستن و برخاستن (30-Second test of up and down) و آزمون زمان‌دار برخاستن و برگشتن (Timed get up and go) براي ارزيابي عملکرد حرکتی استفاده شد. سپس گروه تجربي به مدت 8 هفته در تمرينات پيلاتس (3 جلسه در هفته) شرکت کردند. آزمون ANCOVA جهت تجزیه و تحلیل اطلاعات مورد استفاده قرار گرفت. يافته‌ها: بين دو گروه تجربي و شاهد تفاوت معني‌داري در آزمون 30 ثانیه نشستن و برخاستن از روی صندلی (394/232 = F، 001/0 > P)، آزمون زمان‌دار برخاستن و برگشتن (199/265 = F، 001/0 > P) و آزمون افسردگی (000/92 = F، 001/0 > P) مشاهده شد. نتيجه‌گيري: نتايج تحقيق نشان داد، ورزش پيلاتس در کمک به کاهش سطوح افسردگي و بهبود عملکرد حرکتی مؤثر بوده است.

Title: تأثیر هشت هفته تمرين با توپ فیزیوبال بر اصلاح کیفوز پشتی، تعادل و كيفيت زندگي مردان مبتلا به مصرف مواد مخدر پس از ترك اعتیاد

http://jrrs.mui.ac.ir/index.php/jrrs/article/view/989

مقدمه: ورزش و فعالیت بدنی این پتانسیل را دارند که باعث بهبود و پیشرفت سلامتی و کیفیت زندگی افراد مبتلا به مصرف مواد مخدر شوند. بنابراین هدف تحقیق حاضر، بررسی تأثیر هشت هفته تمرين با توپ فیزیوبال بر اصلاح کیفوز پشتی، تعادل (ایستا و پویا) و كيفيت زندگي مردان مبتلا به مصرف مواد مخدر پس از ترك اعتیاد بود. مواد و روش‌ها: تعداد 30 مرد مبتلا به مصرف مواد مخدر با محدوده سني 20 تا 40 سال که دو ماه از ترک اعتیاد آنان گذشته بود و دارای عارضه افزایش کیفوز پشتی بودند، به صورت در دسترس و هدفمند انتخاب و پس از اخذ رضایت‌نامه به طور تصادفي در دو گروه تجربی (تمرینی) و شاهد قرار داده شدند. زاویه کيفوز آزمودنی‌ها به وسیله خط‌کش منعطف، تعادل پویا توسط آزمون ستاره (Star excursion balance test یا SEBT)، تعادل ایستا با آزمون Romberg و کیفیت زندگی با استفاده از فرم کوتاه پرسش‌نامه کیفیت زندگی (SF-36) اندازه‌گيري گرديد. آزمودنی‌ها به مدت 8 هفته و هر هفته سه جلسه و هر جلسه 60 تا 90 دقیقه تمرينات منتخب را تحت نظر متخصص حرکات اصلاحی و درمانی انجام دادند. جهت تحليل داده‌ها از روش آماري ANOVA براي داده‌هاي تکراري در سطح معنی‌داری 05/0 ≥ P استفاده گردید. يافته‌ها: تغييرات و تعامل در هر سه متغیر تعادل و کیفیت زندگی، در پس‌آزمون نسبت به پیش‌آزمون در گروه تمرینی معنی‌دار بود (05/0 > P). بدین معنی که تمرینات اعمال شده دارای تأثیر مثبتی بوده است. همچنین تفاوت‌های بين گروهي در تمامی متغيرهای تحقيق معنی‌دار نبود (05/0 < P). نتيجه‌گيري: به نظر مي‌رسد برنامه تمرینی با توپ فیزیوبال برای بهبود ناهنجاری کیفوز پشتی، افزایش تعادل و کیفیت زندگی افراد مبتلا به مصرف مواد مخدر در حال ترک مفید است و می‌تواند به عنوان یک روش درمان غیر دارویی در بازتوانی این افراد مؤثر واقع شده و توسط مراکز ترک اعتیاد مورد استفاده قرار گیرد.

Title: مقایسه میزان استفاده از دو استراتژی کل‌خوانی و خواندن آوایی در دانش‌آموزان با افت شنوایی شدید تا عمیق پایه دوم و سوم دبستان شهر اهواز

http://jrrs.mui.ac.ir/index.php/jrrs/article/view/576

مقدمه: بسیاری از کودکان و نوجوانانی که با مشکل شنوایی متولد شده‌اند یا در ماه‌های ابتدایی تولد دچار آسیب‌های شنوایی می‌شوند در آینده مشکلات خواندن و نوشتن را تجربه می‌کنند. هدف از این مطالعه، بررسی و مقایسه میزان استفاده از دو شیوه خواندن آوایی و کل‌خوانی کودکان با افت شنوایی در شهر اهواز بود تا بتواند مقدمه‌ای برای پیشرفت بهتر و صحیح‌تر این کودکان در زمینه خواندن باشد. مواد و روش‌ها: مطالعه حاضر از نوع توصیفی- تحلیلی بود که بر روی 35 دانش‌آموز افت شنوای شدید تا عمیق پایه دوم (تعداد = 23 نفر) و سوم (تعداد = 12 نفر) دبستان از مدارس مخصوص کودکان افت شنوا انجام شد. کل نمونه‌های مورد بررسی در طول 3 ماهه اول سال تحصیلی 91-1390 مورد بررسی قرار گرفتند. جهت انجام این مطالعه، 2 خرده آزمون خواندن کلمات بی‌قاعده و خواندن ناکلمه از آزمون تشخیصی خواندن شیرازی- نیلی‌پور (1384) استفاده گردید. یافته‌ها: دانش‌آموزان پایه دوم دبستان در مهارت کل‌خوانی نسبت به آوایی بهتر عمل کردند و دانش‌آموزان پایه سوم دبستان در مهارت خواندن آوایی بهتر از پایه دوم عمل کردند، ولی یافته‌های به دست آمده از لحاظ آماری معنی‌دار نبود (05/0 ³ P). نتیجه‌گیری: با استفاده از نتایج این مطالعه می‌توان دریافت که پیشرفت مهارت‌های خواندن در کودکان افت شنوا نیز مانند کودکان طبیعی سیر تدریجی دارد. کودکان در ابتدای آموزش خواندن، بیشتر از کل‌خوانی استفاده می‌کنند و با افزایش سن و دانش سوادآموزی، مهارت‌های خواندن به هر دو شیوه در این کودکان پیشرفت می‌کند.

Title: مروری توصیفی بر رویکرد درمانی CO-OP و تأثیر آن بر مشکلات حرکتی کودکان با اختلالات هماهنگی رشدی

http://jrrs.mui.ac.ir/index.php/jrrs/article/view/891

مقدمه: مهارت‌های حرکتی برای بسیاری از فعالیت‌های روزمره کودکان ضروری می‌باشد. این مهارت‌ها برای رشد استقلال در فعالیت‌های مراقبت از خود و فعالیت‌های روزمره دیگر ضروری است و کودکان با مشکلات حرکتی در استقلال مراقبت از خود تأخیر دارند. کاردرمانگران برای بهبود عملکرد حرکتی کودکان از رویکردها و چارچوب مرجع‌های متفاوتی استفاده می‌کنند. یکی از رویکردهای درمانی در دهه‌های اخیر، رویکرد درمانی CO-OP (Cognitive orientation to daily occupational performance) است. رویکرد درمانی CO-OP رویکردی مراجع‌‌مدار است و به صورت انفرادی اجرا می‌گردد که طی استفاده از راهبردهای شناختی، کودک قادر به حل مشکلات کارکرد حرکتی خود خواهد بود. هدف مطالعه حاضر، مروری بر شواهد برای تعیین اثربخشی رویکرد CO-OP در بهبود عملکرد حرکتی کودکان DCD (Developmental coordination disorder) بود. مواد و روش‌ها: در بررسی متون، شواهد پژوهشی با استفاده از جستجوی سیستماتیک در پایگاه‌های اطلاعاتی، Informa، PubMed، Scopus، Google Scholar، Science Direct و ProQuest و مرور منابع لیست شده در مقالات و کتاب‌ها، بر اساس ملاک‌های ورود به مطالعه انتخاب شدند و مورد بررسی قرار گرفتند. یافته‌ها: با توجه به کلید واژه‌های انتخاب شده، تعداد 60 مقاله یافت شد که پس از بررسی اجمالی، تنها 8 مقاله بر اساس ملاک‌های ورود و کناره‌گذاری جهت بررسی بیشتر انتخاب شدند. نتیجه‌گیری: با بررسی مطالعات انجام شده، نشان داده شد که رویکرد درمانی CO-OP می‌تواند به عنوان رویکرد مناسبی در بهبود کارکرد کاری مبتنی بر حرکت کودکان با مشکلات حرکتی مورد استفاده قرار گیرد.
